# ITPR1 Maintains Mitochondrial Redox Homeostasis to Drive Glioblastoma Progression Through Recruitment and Activation of DRP1

**DOI:** 10.3390/antiox15050550

**Published:** 2026-04-26

**Authors:** Shuyan Luo, Mei Tao, Sihan Li, Xingbo Li, Qian Jiang, Quanji Wang, Zihan Wang, Lv Zhou, Kai Shu, Zhuowei Lei, Yimin Huang, Ting Lei

**Affiliations:** 1 Sino-German Neuro-Oncology Molecular Laboratory, Department of Neurosurgery, Tongji Hospital of Tongji Medical College of Huazhong University of Science and Technology, Wuhan 430030, China; d202382230@hust.edu.cn (S.L.); lisihan2019@163.com (S.L.); lxbhusttj@tjh.tjmu.edu.cn (X.L.); d202382335@hust.edu.cn (Q.J.); quanjiwang@tjh.tjmu.edu.cn (Q.W.); zihanwang@hust.edu.cn (Z.W.); m202376657@hust.edu.cn (L.Z.); kshu@tjh.tjmu.edu.cn (K.S.); yimin.huang@tjh.tjmu.edu.cn (Y.H.); 2 Hubei Key Laboratory of Neural Injury and Functional Reconstruction, Huazhong University of Science and Technology, Wuhan 430030, China; 3 Department of Anesthesiology and Pain Medicine, Hubei Key Laboratory of Geriatric Anesthesia and Perioperative Brain Health, Wuhan Clinical Research Center of Geriatric Anesthesia, Tongji Hospital of Tongji Medical College of Huazhong University of Science and Technology, Wuhan 430030, China; meitao1995@tjh.tjmu.edu.cn; 4 Department of Orthopaedics, Tongji Hospital of Tongji Medical College of Huazhong University of Science and Technology, Wuhan 430030, China

**Keywords:** ITPR1, glioblastoma, DRP1, mitophagy, temozolomide, 2-APB

## Abstract

**Background**: Glioblastoma (GBM) exhibits marked cellular heterogeneity and resistance to therapy. Calcium (Ca^2+^) signaling at endoplasmic reticulum (ER)–mitochondria contact sites has emerged as a key regulator of mitochondrial function and cell fate; however, its lineage-specific role and therapeutic relevance in GBM remain unclear. **Methods**: *ITPR1* expression was analyzed using single-cell and bulk RNA sequencing (RNA-seq) datasets and validated by immunohistochemistry and survival analyses. Functional studies were conducted using genetic silencing or CRISPR-mediated activation of *ITPR1*, combined with DRP1 knockdown, Ca^2+^ imaging, transmission electron microscopy, co-immunoprecipitation, mitochondrial fractionation, and mitochondrial functional assays. Therapeutic efficacy was evaluated in orthotopic GBM xenograft models treated with 2-aminoethoxydiphenyl borate (2-APB), temozolomide (TMZ), or their combination. **Results**: ITPR1 was enriched in mesenchymal-like malignant cell states and associated with higher tumor grade, recurrence, and poor prognosis. *ITPR1* knockdown suppressed GBM cell proliferation and tumor growth while promoting intrinsic apoptosis. Mechanistically, loss of ITPR1 impaired ER-to-mitochondria Ca^2+^ transfer, disrupted ER–mitochondria contacts, and altered mitochondrial ultrastructure. This was accompanied by reduced DRP1 Ser616 phosphorylation and mitochondrial recruitment, as well as decreased autophagy and mitophagy activity. Consequently, *ITPR1* knockdown led to mitochondrial depolarization, increased mitochondrial reactive oxygen species (ROS) accumulation, and activation of mitochondria-dependent apoptosis. Conversely, DRP1 knockdown attenuated the mitochondrial and pro-survival effects induced by *ITPR1* overexpression. In vivo, combined treatment with 2-APB and TMZ resulted in greater tumor suppression and prolonged survival compared with either treatment alone, accompanied by increased apoptosis and reduced proliferation in tumor tissues. **Conclusions**: ITPR1 promotes GBM progression by sustaining ER–mitochondria Ca^2+^ coupling and DRP1-dependent mitochondrial quality control, thereby maintaining mitochondrial homeostasis and cell survival. Targeting inositol 1,4,5-trisphosphate receptor (IP_3_R)-mediated Ca^2+^ signaling with 2-APB enhances the therapeutic efficacy of TMZ, suggesting that ITPR1-centered Ca^2+^ signaling may represent a potential therapeutic vulnerability in aggressive GBM.

## 1. Introduction

Glioblastoma (GBM) is the most aggressive primary brain tumor in adults, with a median survival of only 14–18 months despite aggressive treatments involving surgery, radiotherapy, and temozolomide (TMZ) chemotherapy [1]. The significant challenges in treating GBM stem from profound inter- and intra-tumoral heterogeneity, metabolic plasticity, and therapy resistance [2,3]. As a result, metabolic adaptation and mitochondrial remodeling have become key drivers of GBM progression and therapeutic failure. [3,4].

In recent years, oxidative stress has been increasingly recognized as a key regulator of GBM progression and resistance to treatment [5]. These processes are tightly regulated by various molecular mechanisms that influence mitochondrial dynamics and redox balance. Recent studies have shown that histone modifications, such as p300-mediated H3K18 lactylation, can promote mitochondrial reactive oxygen species (ROS) accumulation through inhibiting mitophagy, thereby enhancing the efficacy of therapeutic agents like dopamine agonists in prolactinomas [6]. This mechanism further emphasizes the importance of mitochondrial ROS in disease progression and therapeutic response. The mitochondria, as the primary site for ROS production, play a pivotal role in regulating cellular metabolism and apoptosis [7]. Notably, the maintenance of mitochondrial bioenergetics and redox balance is closely regulated by calcium (Ca^2+^) signaling, which controls the transfer of Ca^2+^ from the endoplasmic reticulum (ER) to the mitochondria [8,9]. At the mitochondria-associated membranes (MAMs), key ER-localized Ca^2+^ channels, such as the inositol 1,4,5-trisphosphate receptor (IP_3_R) family, regulate mitochondrial oxidative phosphorylation, ROS production, and apoptotic susceptibility [10].

Dysregulated ER–mitochondria Ca^2+^ coupling has been linked to multiple pathological conditions, including both cancer and neurological disorders [7,11,12]. Notably, ITPR1 is highly expressed in the central nervous system, particularly in cerebellar Purkinje neurons, where it plays a critical role in intracellular Ca^2+^ signaling. Loss-of-function mutations in ITPR1 have been associated with neurological diseases such as spinocerebellar ataxia [13]. At mitochondria-associated membranes (MAMs), IP_3_Rs form microdomains with voltage-dependent anion channels (VDACs) and other scaffold proteins to control Ca^2+^ uptake into mitochondria, thereby regulating oxidative phosphorylation, reactive oxygen species (ROS) production, and apoptosis [11]. However, the role of specific IP_3_R isoforms, particularly ITPR1, in regulating mitochondrial dynamics, oxidative stress, and therapeutic resistance in GBM remains insufficiently explored. This study focuses on ITPR1 as a central player in the regulation of mitochondrial ROS and oxidative stress, processes that in turn modulate GBM progression and resistance to chemotherapy. Beyond cancer, dysregulation of ITPR1-mediated Ca^2+^ signaling and mitochondrial ROS control has also be associated with neurological disorders, suggesting potential relevance to other diseases [14,15,16].

Recent advances in single-cell transcriptomic profiling have further highlighted the profound cellular heterogeneity of GBM, revealing distinct transcriptional states that reflect lineage hierarchies and microenvironmental adaptation. In this context, GBM neoplastic cells can be organized into four transcriptionally distinct states: oligodendrocyte progenitor cell-like (OPC-like), neural progenitor cell-like (NPC-like), astrocyte-like (AC-like), and mesenchymal-like (MES-like), which reflect dynamic lineage hierarchies and microenvironmental adaptation [17]. MES-like and AC-like populations are associated with heightened inflammatory signaling, invasive behavior, and poor clinical outcomes [17]. Among these, the MES-like state is strongly associated with inflammatory signaling, invasive behavior, therapeutic resistance, and poor clinical outcomes. Yet the upstream signaling pathways that maintain MES-like metabolic fitness and survival advantages are not fully understood.

This study suggests that ITPR1 is selectively enriched in the MES-like malignant state and may be associated with poor clinical outcomes in GBM patients. Mechanistically, ITPR1 maintains ER–mitochondria Ca^2+^ coupling and preserves mitochondrial integrity by promoting DRP1 activation and mitochondrial fission, thereby sustaining mitophagy and limiting ROS-mediated intrinsic apoptosis. Genetic and pharmacological inhibition of ITPR1 disrupts mitochondrial homeostasis, suppresses tumor growth, and enhances the therapeutic efficacy of TMZ in orthotopic GBM models. These findings suggest that ITPR1 may serve as a critical regulator of mitochondrial dynamics and a potential therapeutic target in aggressive GBM.

## 2. Materials and Methods

### 2.1. Patient Datasets

RNA-sequencing data (RNA-seq) and corresponding clinical information for GBM patients (WHO: IV) were obtained from The Cancer Genome Atlas (TCGA) and Chinese Glioma Genome Atlas (CGGA; 693 and 325 cohorts) databases [18,19]. Publicly available scRNA-seq datasets of human GBM samples were downloaded from CellXGene [20]. Neoplastic cells were identified based on the annotated information and subsequently classified into OPC-like, NPC-like, AC-like, and MES-like states using previously established meta-programs or module scores [17].

### 2.2. Gene Set Scoring

The meta-program gene list was extracted from the published research [17] (Supplementary Table S1). The CRG score for each GBM patient from the TCGA and CGGA databases was calculated by the ssGSEA method of the GSVA package (version 1.50.0). The CRG scores and MES-like, AC-like, NPC-like, and OPC-like scores were evaluated at the single-cell level by AUCell (version 1.24.0) analyses.

### 2.3. Cell Culture and Transfection

The human glioma cell lines (LN229 and U251) used in this study were obtained from the American Type Culture Collection (ATCC, Manassas, VA, USA). The human cell lines LN229 and U251 correspond to ATCC^®^ CRL-2611™ and ATCC^®^ HTB-17™, respectively. These cell lines are commercially available, and their genetic background information is maintained in the ATCC database. Human GBM cell lines (LN229, U251) were cultured in DMEM supplemented with 10% FBS. GSCs were provided by Professor Jianghong Man (State Key Laboratory of Proteomics, National Center for Biomedical Analysis, Beijing, China), derived from primary GBM samples or patient-derived GBM xenografts. GSCs were cultured in Neurobasal-A medium (Gibco, Thermo Fisher Scientific, Waltham, MA, USA) with B27, 10 ng/mL EGF (Gibco, Thermo Fisher Scientific, Waltham, MA, USA), 10 ng/mL bFGF (Gibco, Thermo Fisher Scientific, Waltham, MA, USA), 1 mM sodium pyruvate (Gibco, Thermo Fisher Scientific, Waltham, MA, USA), and 2 mM L-glutamine (Gibco, Thermo Fisher Scientific, Waltham, MA, USA). For differentiation into GDCs, GSCs were cultured in DMEM (high glucose) supplemented with 1% L-glutamine, 1% penicillin/streptomycin, and 10% FBS. Complete media were adjusted to resemble Neurobasal-A, containing 0.4 mM serine and 0.4 mM glycine. For serine and glycine deprivation, SG media were used (serine and glycine omitted). For rescue experiments, +SG media were used (0.8 mM serine and 0.8 mM glycine).

*ITPR1* knockdown was achieved using a CRISPR/Cas9 inhibition system. *ITPR1* overexpression was induced using a CRISPR/Cas9 activation system. DRP1 knockdown was performed with specific siRNAs, and *MT1X* knockdown was established using shRNA. Stable cell lines were generated by lentiviral transduction. The sequence of the sgRNA, shRNA, or siRNA is listed in Supplementary Table S2.

### 2.4. Ca^2+^ Signaling Analysis

LN229 cells were seeded in 24-well plates and treated with 5 µM Fluo-4 AM (Thermo Fisher, Waltham, MA, USA), Rhod-2 AM (Yeasen Biotechnology, Shanghai, China), or Mag-Fluo-4 AM (AAT Bioquest, Sunnyvale, CA, USA) in Hank’s balanced salt solution (HBSS) (Gibco, Thermo Fisher Scientific, Waltham, MA, USA). After incubation for 30–60 min at 37 °C and 5% CO_2_ in the dark, cells were washed twice with HBSS and further incubated for 30 min at room temperature. Fluorescence images were captured using an inverted fluorescence microscope (OLYMPUS-CKX53, Olympus Corporation, Tokyo, Japan), with excitation/emission wavelengths of 494/516 nm for Fluo-4 AM, 549/578 nm for Rhod-2 AM, and 494/516 nm for Mag-Fluo-4 AM. Fluorescence intensity was quantified using ImageJ (version 1.53t, National Institutes of Health, Bethesda, MD, USA).

### 2.5. Western Blot Analysis

Cells were lysed in RIPA buffer (Millipore, Burlington, MA, USA) containing 1% PMSF (Sigma-Aldrich, St. Louis, MO, USA) and 1% protease inhibitor cocktail (MedChemExpress, Monmouth Junction, NJ, USA). Protein concentration was determined using the BCA assay (Sigma-Aldrich, St. Louis, MO, USA). Denatured proteins were separated by SDS-PAGE, transferred to PVDF membranes, and then blocked. Membranes were incubated overnight at 4 °C with primary antibodies specific to ITPR1 (1:1000; Santa Cruz Biotechnology, Dallas, TX, USA, #sc-271197), Tubulin (1:1000; ABclonal Biotechnology, Wuhan, China, #A12289), Cleaved Caspase-3 (1:1000; Cell Signaling Technology, Danvers, MA, USA, #9661), Caspase 3 (1:1000; Cell Signaling Technology, Danvers, MA, USA, #9662), BAX (1:1000; CST, #2772), BCL-2 (1:1000; CST, #3498), p-DRP1^Ser616^ (1:1000; ABclonal Biotechnology, Wuhan, China, #AP1573), DRP1 (1:3000; ABclonal Biotechnology, Wuhan, China, #A21968), SDHA (1:1000; ABclonal Biotechnology, Wuhan, China, #A13852), Lamp1 (1:1000; ABclonal Biotechnology, Wuhan, China, #A21194), p62 (1:1000; Abclonal, #A19700), ATG5 (1:1000; ABclonal Biotechnology, Wuhan, China, #A18677), and LC3B (1:1000; ABclonal Biotechnology, Wuhan, China, #A19665). After washing, membranes were incubated with HRP-conjugated secondary antibody for 1 h at room temperature. Protein bands were detected using a chemiluminescent substrate (Sparkjade, Shandong, China).

### 2.6. Immunohistochemistry (IHC)

The paraffin-embedded tissue sections were deparaffinized in xylene, followed by rehydration through a graded ethanol series, and then subjected to antigen retrieval using pH 9.0 EDTA buffer in a microwave. Endogenous peroxidase activity was blocked with 3% hydrogen peroxide. The sections were then incubated overnight at 4 °C with primary antibodies against ITPR1 (1:500; Santa Cruz Biotechnology, Dallas, TX, USA, #sc-271197) and Ki-67 (1:5000; Proteintech Group, Inc., Rosemont, IL, USA, #27309-1-AP). Apoptosis was detected using the TUNEL staining kit (G1507, Servicebio, Wuhan, China) following the manufacturer’s protocol. The sections were incubated with a secondary antibody at room temperature for 1 h. DAB staining was performed, and the reaction was stopped in distilled water. Hematoxylin staining was applied, followed by dehydration, clearing in xylene, and mounting with neutral balsam. Immunoreactivity was assessed by scoring staining intensity (0–3) and the proportion of positive cells (<5% = 0, 5–25% = 1, 26–50% = 2, 51–75% = 3, >76% = 4). The Immunoreactive Score (IRS) was calculated as the product of staining intensity and positive cell score. Scores ≤ 6 indicated low expression, while scores > 6 indicated high expression.

### 2.7. Co-Immunoprecipitation

Cells were collected and lysed using a buffer containing 0.5% Triton X-100, 50 mM Tris-Cl (pH 8.0), 150 mM NaCl, and 1 mM EDTA. The resulting protein lysates were incubated with the ITPR1 antibody (5 μg, Santa Cruz Biotechnology, Dallas, TX, USA, #sc-271197) overnight. On the following day, 50 μL of Protein A/G agarose beads were added, and the mixture was rotated at 4 °C for 3–4 h with gentle agitation. Beads were then washed three times with TBST. Immunoprecipitated proteins were finally eluted from the beads with 2× SDS-PAGE loading buffer and analyzed by Western blotting.

### 2.8. Immunofluorescence and Colocalization Analysis

Cells were fixed with 4% paraformaldehyde, permeabilized with 0.2% Triton X-100, and blocked with 5% BSA. Primary antibodies against DRP1 and TOMM20 were incubated overnight at 4 °C, followed by appropriate fluorescent secondary antibodies. Nuclei were counterstained with DAPI. Images were acquired using a confocal laser scanning microscope (LSM 900 Airyscan, Zeiss, Oberkochen, Germany). Colocalization between DRP1 and TOMM20 was quantified using ImageJ with the JACoP plugin [21]. Manders’ colocalization coefficient was calculated from at least five randomly selected fields per sample.

### 2.9. ER–Mitochondria Colocalization Analysis

To assess ER–mitochondria contact, live cells were stained with ER-Tracker Red (Yeasen Biotechnology, Shanghai, China, #40764ES20) and MitoTracker Green (Yeasen Biotechnology, Shanghai, China, #40742ES50) according to the manufacturer’s instructions. Confocal images were acquired under identical settings across groups. Colocalization analysis was performed using ImageJ, and Pearson’s correlation coefficient was calculated from at least five random fields per experiment.

### 2.10. Mitochondrial Morphology Analysis

Cells were stained with MitoTracker Green and imaged by confocal microscopy. Mitochondrial morphology was analyzed using the MiNA (Mitochondrial Network Analysis) [22] toolset in ImageJ. Mean branch length was used as an indicator of mitochondrial network connectivity and fragmentation. For each condition, five randomly selected fields were analyzed per experiment, with three independent experiments performed.

### 2.11. Liquid Chromatography–Tandem Mass Spectrometry (LC-MS/MS)

Following lysis with lysis buffer, cellular proteins were incubated overnight with either the ITPR1 antibody (5 μg, Santa Cruz Biotechnology, Dallas, TX, USA, #sc-271197) or control IgG (5 μg; Cell Signaling Technology, Danvers, MA, USA, #68860). Protein A/G agarose beads were then added and incubated for an additional 3–4 h. After immunoprecipitation, proteins were subjected to extraction, enzymatic digestion, and peptide desalting to obtain peptide solutions suitable for mass spectrometry analysis. Peptide samples were analyzed using a liquid chromatography–tandem mass spectrometry (LC-MS/MS) platform. This technique enables high-resolution and rapid detection of target peptides. In the first mass spectrometry stage (MS1), the mass-to-charge ratios (*m*/*z*) of intact peptides were measured. Fragmented peptide ions were subsequently analyzed in the second stage (MS2), and their m/z values were used to infer amino acid sequences. The resulting mass spectrometry data were searched against a protein database for protein identification. Differentially expressed proteins between the ITPR1 antibody group and the IgG control group were identified based on the criteria FC > 1.5 and *p* < 0.05. Proteins meeting both differential expression thresholds and the peptide quality requirements (unique peptides ≥ 2 and score > 30) were considered differentially enriched proteins. These proteins, together with ITPR1, were further subjected to interaction prediction analysis using the STRING database.

### 2.12. Molecular Docking

The molecular structure files of ITPR1 (PDB ID: 1N4K) and DRP1 (PDB ID: 9N7Z) were downloaded from the Protein Data Bank (PDB). Rigid-body docking was performed using the GRAMM platform, in which both the ligand and receptor proteins are treated as rigid structures without conformational changes. The algorithm scans the receptor surface to identify optimal geometric complementarities with the ligand protein. A total of 10 valid docking conformations were generated by GRAMM [23]. Among them, the top-ranked model exhibited the most favorable binding characteristics, with a predicted binding energy of –7.9 kcal/mol. Following docking, binding free energy was calculated using PDBePISA (European Bioinformatics Institute, Cambridge, UK), and the docking interface was visualized using PyMOL (version 3.1, Schrödinger, New York, NY, USA).

### 2.13. CCK8, Colony Formation and Edu Assays

For the cell viability assay, 2000 cells were seeded in each well of a 96-well plate. Viability was measured on specified days using the CCK8 kit (Yeasen Biotechnology, Shanghai, China) according to the manufacturer’s instructions. Colony formation assays were performed by seeding cells at low density and staining colonies after 10–14 days. Cell proliferation was assessed with either EdU imaging kits (Yeasen Biotechnology, Shanghai, China), following the provided protocols. These experiments were performed in triplicate to ensure reliable data.

### 2.14. Apoptosis Assay

Apoptosis was assessed using an Annexin V-APC/7-AAD apoptosis kit (Elabscience Biotechnology Co., Ltd., Wuhan, China) according to the manufacturer’s protocol and analyzed by flow cytometry. A total of 10,000 cells per sample were examined, and data analysis was performed using FlowJo software (version 10.8, BD Biosciences, Ashland, OR, USA).

### 2.15. Measurement of Mitochondrial ROS

Mitochondrial superoxide was detected using MitoSOX Red (Thermo Fisher Scientific, Waltham, MA, USA), and cells were treated with 5 μM MitoSOX Red and 50 nM MitoTracker Green (Yeasen Biotechnology, Shanghai, China) for 30 min at 37 °C. Intracellular ROS levels were measured by flow cytometry using 10 μM 2′,7′-dichlorodihydrofluorescein diacetate (DCFH-DA) (Sigma-Aldrich, St. Louis, MO, USA) for 20 min at 37 °C. Fluorescence images were captured using an inverted fluorescence microscope (OLYMPUS-CKX53). The excitation/emission wavelengths for MitoSOX Red were 510/580 nm and for DCFH-DA were 488/525 nm. The intracellular ROS analysis was performed using FlowJo 10.8 software.

### 2.16. Mitochondrial Transmembrane Potential (ΔΨm) Assay

The mitochondrial membrane potential (MMP) was assessed using the fluorescent probe JC-1 (BD Pharmingen, BD Biosciences, San Jose, CA, USA). The JC-1 working solution was added to the culture medium and incubated at 37 °C for 30 min. After two washes with cold staining buffer and centrifugation, the cells were resuspended in the staining buffer. MMP quantification was performed by flow cytometry (BD), with mitochondrial fluorescence detected at an excitation wavelength of 488 nm. The red fluorescence from JC-1 aggregates was analyzed in the PE channel, and the green fluorescence from JC-1 monomers was analyzed in the FITC channel. The MMP analysis was performed using FlowJo 10.8 software.

Mitochondrial membrane potential (ΔΨm) was assessed using tetramethylrhodamine methyl ester (TMRM) (Yeasen Biotechnology, Shanghai, China, #732773ES08). Cells were incubated with TMRM (100 nM) for 30 min at 37 °C and then imaged by confocal microscopy. Fluorescence intensity was quantified using ImageJ and normalized to control groups. At least five fields per sample were analyzed in each experiment, with three independent experiments performed.

### 2.17. Transmission Electron Microscopy and MAM Quantification

Cells were fixed in 2.5% glutaraldehyde and post-fixed in 1% osmium tetroxide, followed by dehydration through a graded ethanol series and embedding in epoxy resin. Ultrathin sections (70–90 nm) were cut and imaged using a transmission electron microscope (HT7800, Hitachi High-Tech Corporation, Tokyo, Japan).

For analysis of mitochondria-associated membranes (MAMs), TEM images were processed using ImageJ. The ER–mitochondria contact sites were manually outlined in two-dimensional sections. MAMs were defined as regions where the distance between the ER and mitochondrial membranes was ≤30 nm. The following parameters were quantified: MAM density (total MAM length per mitochondrial perimeter), number of MAMs per mitochondrion, MAM length, and the percentage of mitochondrial surface in close apposition to the ER. At least 20 mitochondria per cell and 10 cells per condition were analyzed in each independent experiment.

### 2.18. Orthotopic Xenograft

All animal experiments conducted in this study were approved by the Institutional Review Board of Tongji Hospital, Tongji Medical College of Huazhong University of Science and Technology (Approval No. TJH-24-07-046). Sample sizes were as follows: genetic orthotopic xenograft experiments, *n* = 6 mice per group; orthotopic drug treatment study, *n* = 6 mice per group (vehicle, 2-APB, TMZ, and combination). Animals were randomly assigned to experimental groups using a random number generator. Investigators were blinded to group allocation during tumor measurement, imaging analysis, and histological evaluation. No statistical methods were used to predetermine sample size. Sample sizes were chosen based on prior experience with similar models and were sufficient to detect biologically relevant differences. No animals or data points were excluded from the analysis. As previously described, an orthotopic GBM xenograft model was established via intracranial implantation of GSCs [24]. GSC19-luciferase cells were implanted orthotopically into nude mice. After 7 days, the mice were randomly assigned to a vehicle, 2-APB (Selleck Chemicals, Houston, TX, USA, #S6657), temozolomide (TMZ) (Selleck Chemicals, Houston, TX, USA, #S1237), or combination treatment group. The 2-APB group of mice was treated with intraperitoneal injection of 2-APB (10 mg kg^−1^) every day for a total of 20 days. The TMZ group of mice was treated with intraperitoneal injections of TMZ (30 mg/kg) every other day for a total of 20 days. Tumor growth was monitored by in vivo bioluminescence imaging where applicable. Survival was analyzed by Kaplan–Meier curves with log-rank tests. Brains were harvested for H&E staining and histologic assessment of tumor burden.

### 2.19. Statistical Analysis

Data are presented as mean ± SEM from at least three independent experiments. For comparisons between two groups, normality was tested using the Shapiro–Wilktest. If the data were normally distributed, a two-tailed unpaired Student’s *t*-test was applied; otherwise, the Mann–Whitney U test was used. For comparisons among multiple groups, normality and homogeneity of variances were first assessed, followed by one-way ANOVA with Tukey’s post hoc test for pairwise comparisons. Kaplan–Meier survival curves were compared using the log-rank test. Correlations were determined using Pearson’s correlation coefficient. A *p* value of <0.05 was considered statistically significant. Statistical analyses were performed using R (version 4.3.1, R Foundation for Statistical Computing, Vienna, Austria) or GraphPad Prism (version 10.0, GraphPad Software, San Diego, CA, USA).

## 3. Results

### 3.1. Elevated ITPR1 Expression in Mesenchymal-like GBM and Predicts Poor Clinical Outcome

**Figure 1:**
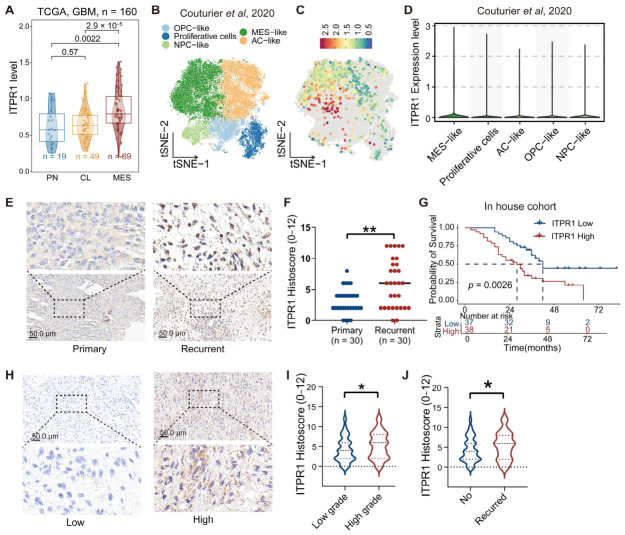
ITPR1 is preferentially expressed in the MES-like malignant state and correlates with tumor aggressiveness and poor prognosis in GBM. (**A**) t-SNE plot showing the neoplastic subclusters in the GBM scRNA-seq dataset (Couturier et al., 2020) [26], defined by meta-program. (**B**) t-SNE plot illustrating ITPR1 expression in neoplastic subclusters. (**C**) Violin plots showing ITPR1 expression across GBM transcriptome subtypes. (**D**) Violin plot showing ITPR1 expression of GBM patients across different neoplastic subcellular clusters defined by the malignant meta-program subtypes in the TCGA cohort. (**E**) Representative IHC images showing ITPR1 expression in primary and recurrent GBM tissues (scale bar: 50 μm). (**F**) Quantitative analysis of ITPR1 expression in primary and recurrent GBM tissues by IHC. (**G**) Kaplan–Meier survival analysis for high and low ITPR1 expression in high-grade GBM patients. (**H**) Representative IHC images of ITPR1 expression levels in low and high-grade GBM tissues (scale bar: 50 μm). (**I**) Statistical analysis of ITPR1 expression in low-grade and high-grade GBM tumor tissues. (**J**) Statistical analysis of ITPR1 expression in recurrent and non-recurrent GBM tumor tissues. (* *p* < 0.05, ** *p* < 0.01).

Neoplastic cells in glioblastoma (GBM) can be classified into four canonical malignant cellular states: oligodendrocyte progenitor cell-like (OPC-like), neural progenitor cell-like (NPC-like), astrocyte-like (AC-like), and mesenchymal-like (MES-like), consistent with previously defined cellular hierarchies [17] (Figure 1A and Figure S1A–C). The IP_3_R family, comprising ITPR1, ITPR2, and ITPR3, encodes endoplasmic reticulum (ER)-localized Ca^2+^ release channels that mediate Ca^2+^ transfer from the ER to mitochondria [11]. Analysis of the single-cell RNA sequencing (scRNA-seq) dataset revealed that ITPR1 was preferentially expressed in MES-like malignant cell clusters, which were associated with elevated inflammatory activity, increased invasive potential, and poorer clinical prognosis compared with other transcriptional states [25] (Figure 1B,C). Consistently, bulk RNA-seq data from the TCGA and CGGA cohorts further demonstrated that ITPR1 expression was significantly upregulated in MES-subtype tumors (Figure 1A and  Figure S1D,E).

To validate the expression of ITPR1 in GBM tumor tissues, we analyzed ITPR1 expression in relation to clinical features (Table 1). The recurrence rates and WHO grade significantly differed between ITPR1 high and low expression groups, while no significant differences were observed in terms of gender or age. Notably, ITPR1 expression was significantly upregulated in both primary and recurrent GBM tumor tissues (Figure 1E,F). Survival analysis revealed that high ITPR1 expression in high-grade glioma patients was associated with poorer survival outcomes compared to patients with low ITPR1 expression (Figure 1G). Additionally, ITPR1 expression was found to be higher in high-grade and recurrent glioma tumors (Figure 1H,J).

### 3.2. ITPR1 Promotes GBM Cell Proliferation and Tumor Growth by Suppressing Apoptosis

**Figure 2:**
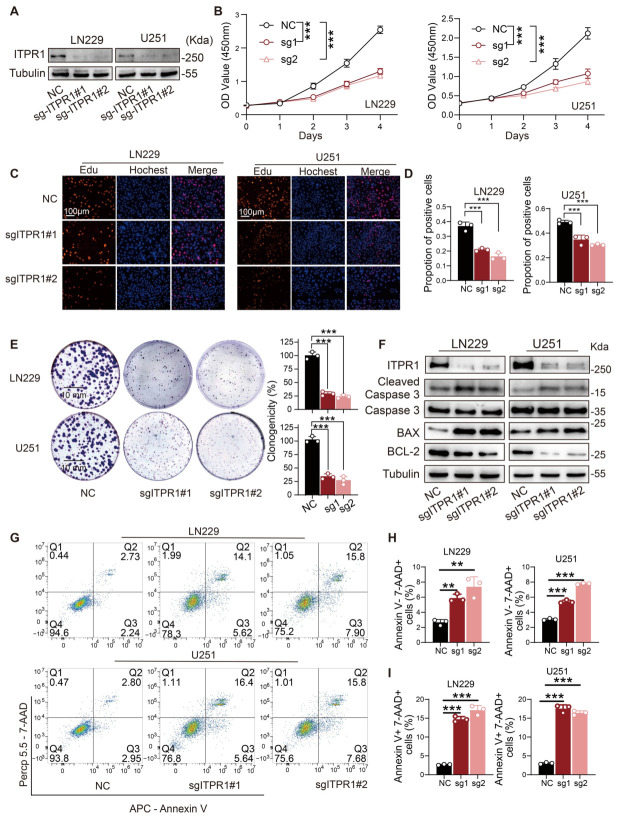
Knockdown suppresses GBM cell proliferation and induces apoptosis in vitro. (**A**) Western blot analysis of ITPR1 expression in LN229, U251, and U87 cells. (**B**) CCK8 assay showing a significant decrease in cell viability in LN229 and U251 cell lines after *ITPR1* knockdown. (**C**) Representative images of EdU incorporation assays demonstrating reduced cell proliferation in LN229, U251, and U87 cells after *ITPR1* knockdown (scale bar: 100 μm). (**D**) Quantification of the EdU incorporation assay showing a significant decrease in proliferating cells following *ITPR1* knockdown in LN229, U251, and U87 cells. (**E**) Colony formation assay revealing reduced colony formation ability of LN229 and U251 cell lines upon *ITPR1* knockdown (scale bar: 10 mm). Quantification of colony numbers is shown on the right. (**F**) Western blot analysis of apoptosis-related proteins in LN229 and U251 cell lines after ITPR1 knockdown. Increased cleaved caspase-3 and BAX expression and decreased BCL-2 expression was observed after *ITPR1* knockdown. (**G**) Representative flow cytometry images showing increased apoptosis in LN229 and U251 cell lines upon *ITPR1* knockdown. (**H**) Flow cytometry analysis of Annexin V and 7-AAD staining showing increased apoptosis in LN229 and U251 cell lines after *ITPR1* knockdown. (**I**) Quantification of the percentage of apoptotic cells (Annexin V+/7-AAD+ cells) in LN229 and U251 cell lines following *ITPR1* knockdown. Black represents the negative control (NC) group, red represents the sg-ITPR1#1 group, and pink represents the sg-ITPR1#2 group. (** *p* < 0.01, *** *p* < 0.001).

To explore the functional role of ITPR1 in GBM, we investigated its impact on tumor cell growth and proliferation ability. *ITPR1* knockdown significantly inhibited cell viability and proliferation, as demonstrated by CCK8 assays, EdU incorporation assays, and colony formation assays (Figure 2A–E). These results indicate that ITPR1 is crucial for maintaining GBM cell proliferation. Next, we examined the effect of *ITPR1* knockdown on the apoptosis process. Western blot analysis revealed an increase in cleaved caspase-3 and BAX levels, alongside a decrease in BCL-2 expression, indicating the induction of apoptosis upon ITPR1 silencing (Figure 2F). Then, we further confirmed that *ITPR1* knockdown promoted apoptosis in GBM cells, as evidenced by an increased percentage of both early and late apoptotic cells (Figure 2G–I).

**Figure 3:**
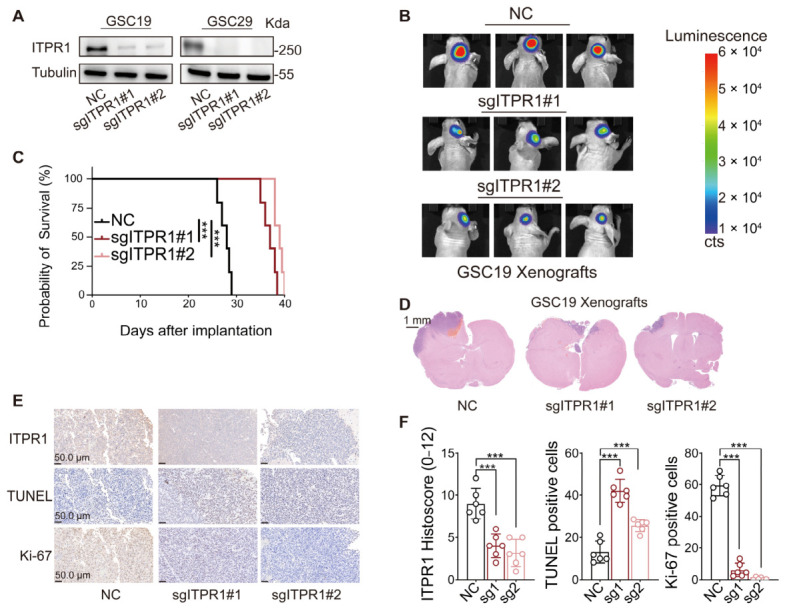
*ITPR1* knockdown impairs tumor growth and prolongs survival in orthotopic GBM xenograft models. (**A**) Western blot analysis of ITPR1 expression in GSC19 and GSC29 cells. (**B**) Representative bioluminescence imaging of orthotopic tumor transplantation in the NC and *ITPR1* knockdown groups, monitored at different time points. (**C**) Kaplan–Meier survival analysis of nude mice (transplanted with control (NC) or *ITPR1* knockdown GSC19 cells into their brains). (**D**) Representative images of H&E-stained sections of mouse brains after GSC19 transplantation. (**E**) Representative IHC images showing ITPR1, TUNEL, and Ki-67 expression in control and ITPR1-knockdown GSC19 xenografts. (**F**) Immunohistochemical staining showing increased TUNEL+ apoptotic cells and reduced Ki-67+ proliferating cells in GSC19 tumors with *ITPR1* knockdown (scale bar: 50 μm). For in vivo experiments (**B**–**F**), each group included *n* = 6 mice. (*** *p* < 0.001).

To evaluate the impact of ITPR1 on tumorigenesis, we generated orthotopic GBM xenografts by implanting control or ITPR1-knockdown GSCs into the brains of immunodeficient mice. *ITPR1* knockdown significantly resulted in smaller tumors and prolonged mouse survival, as demonstrated by bioluminescence imaging and H&E staining (Figure 3A–D). Furthermore, IHC analysis of tumor tissues from orthotopic xenografts revealed increased apoptosis and reduced cell proliferation in tumors with *ITPR1* knockdown (Figure 3E,F). These results suggest that ITPR1 silencing might impair tumor growth and progression by inducing apoptosis and reducing proliferation in GBM xenografts.

### 3.3. ITPR1 Maintains ER–Mitochondria Ca^2+^ Coupling and Regulates Mitochondrial Fission Through DRP1

**Figure 4:**
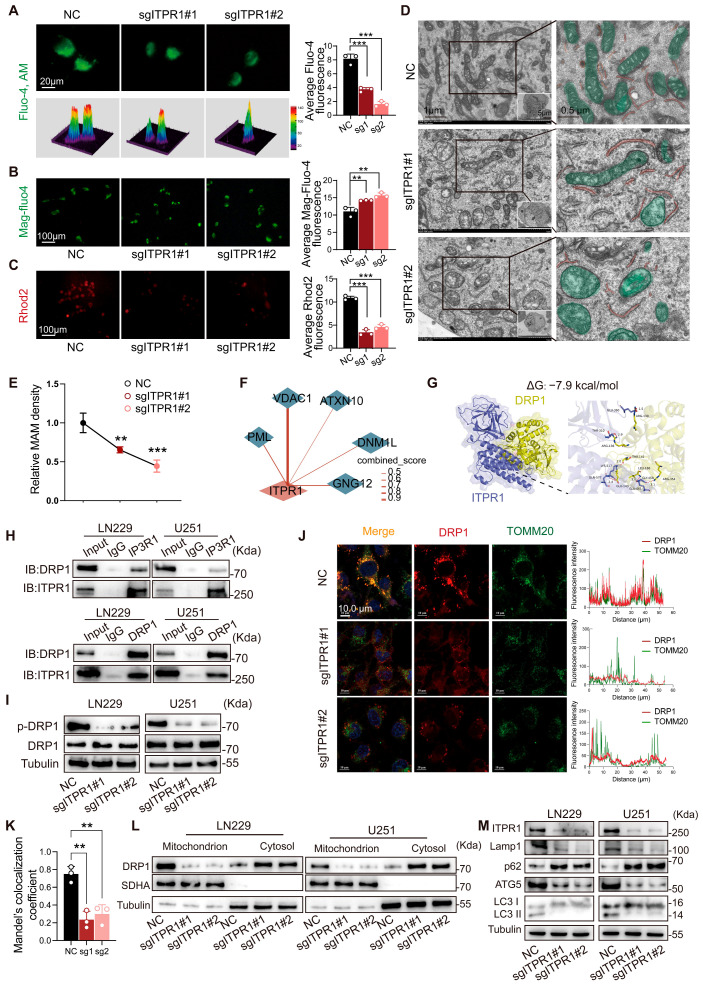
ITPR1 maintains ER–mitochondria Ca^2+^ transfer and promotes DRP1-dependent mitochondrial fission and mitophagy. (**A**) Representative Fluo-4 AM fluorescence images (cellular calcium probe), including 3D surface plots and intensity analysis, demonstrating that ITPR1-knockdown decreases cytosolic Ca^2+^ levels, including 3D surface plots and intensity analysis, demonstrating that ITPR1-knockdown decreases cytosolic Ca^2+^ levels (scale bar: 20 μm). (**B**) Representative Mag-Fluo4 fluorescence images (ER calcium probe), with intensity analysis showing that ITPR1-knockdown slightly increases ER Ca^2+^ storage (scale bar: 100 μm). (**C**) Representative Rhod2 fluorescence images (mitochondrial calcium probe), with intensity analysis showing that *ITPR1* knockdown reduces mitochondrial Ca^2+^ levels (scale bar: 100 μm). (**D**) Transmission electron microscopy (TEM) images illustrating the disruption of ER–mitochondrial contact sites and altered mitochondrial morphology upon ITPR1-knockdown (left scale bar: 1 μm; right scale bar: 0.5 μm). (**E**) Quantitative statistical analysis of MAM density in different groups of LN229 cells. (**F**) Liquid chromatography–tandem mass spectrometry (LC-MS/MS) analysis of ITPR1 immunoprecipitation combined with STRING protein-interaction network mapping identifies DNM1L/DRP1 as one of the high-confidence ITPR1-interacting proteins. (**G**) Molecular docking analysis using the murine ITPR1 structure predicts a stable interaction between ITPR1 and DRP1 in murine species (binding energy: −7.9 kcal/mol). In the docking illustration, purple represents the ITPR1 protein two-dimensional structure, and yellow represents the DRP1 protein two-dimensional structure. (**H**) Co-immunoprecipitation validation of endogenous ITPR1-DRP1 interaction in GBM cells. (**I**) Western blot showing decreased DRP1 phosphorylation at Ser616 upon *ITPR1* knockdown. (**J**) Immunofluorescence analysis of DRP1 localization relative to mitochondria in LN229 cells under control (NC) and *ITPR1* knockdown (sgITPR1#1 and sgITPR1#2) conditions. Scale bar: 10 μm. Line-scan analysis (right panels) shows the fluorescence intensity profiles of DRP1 and TOMM20 along the indicated regions. (**K**) Quantification of DRP1 and TOMM20 colocalization using Manders’ colocalization coefficient. (**L**) Western blot analysis of mitochondrial fractionation showing reduced DRP1 recruitment to mitochondria in LN229 and U251 cell lines after *ITPR1* knockdown. (**M**) Western blot analysis showing decreased autophagy and mitophagy marker levels upon *ITPR1* knockdown. (** *p* < 0.01, *** *p* < 0.001).

Membrane contact sites facilitate direct communication between intracellular organelles, among which the endoplasmic reticulum (ER) forms specialized interfaces with mitochondria known as mitochondrial-associated membranes (MAMs) [27]. Given that ITPR1 is predominantly localized to the ER membrane and functions as a major Ca^2+^ release channel, we next examined whether ITPR1 regulates mitochondrial dynamics through ER–mitochondria coupling. Fluorescence imaging using organelle-specific Ca^2+^ probes revealed that *ITPR1* knockdown significantly reduced cytosolic Ca^2+^ levels, as assessed by Fluo-4 AM staining (Figure 4A). In contrast, ER Ca^2+^ levels, measured by Mag-Fluo-4, were modestly increased (Figure 4B), while mitochondrial Ca^2+^ levels, detected by Rhod-2, were markedly decreased (Figure 4C). These findings suggest impaired Ca^2+^ transfer from the ER to mitochondria following *ITPR1* knockdown. Consistent with this, transmission electron microscopy (TEM) revealed increased ER–mitochondria distance and disrupted ultrastructural organization in *ITPR1*-deficient cells (Figure 4D). Quantitative analysis further demonstrated a significant reduction in MAM density (Figure 4E). Additional morphometric analysis based on two-dimensional tracing confirmed a decrease in MAM number, length, and mitochondrial apposition to the ER (Supplementary Figure S2A,B). Moreover, live-cell confocal imaging using ER-Tracker and MitoTracker showed reduced ER–mitochondria colocalization upon *ITPR1* knockdown (Supplementary Figure S2C,D), supporting a loss of ER–mitochondria contact integrity.

To investigate the molecular mechanisms underlying these alterations, we performed immunoprecipitation followed by LC-MS/MS analysis to identify ITPR1-interacting proteins. Among the high-confidence candidates, DNM1L (DRP1), a key regulator of mitochondrial fission, was identified (Figure 4F and Supplementary Table S3). Molecular docking analysis predicted a stable interaction between ITPR1 and DRP1 (binding energy: −7.9 kcal/mol) (Figure 4G), which was further validated by co-immunoprecipitation in GBM cells (Figure 4H). Functionally, *ITPR1* knockdown led to a marked decrease in DRP1 phosphorylation at Ser616 (Figure 4I), a modification required for DRP1 activation and mitochondrial fission. Consistent with this, immunofluorescence analysis showed reduced colocalization of DRP1 with the mitochondrial marker TOMM20 (Figure 4J,K), indicating impaired mitochondrial recruitment of DRP1. This finding was further supported by mitochondrial fractionation assays, which confirmed decreased DRP1 abundance in mitochondrial fractions following *ITPR1* knockdown (Figure 4L).

Given the central role of DRP1 in mitochondrial dynamics, we next assessed mitochondrial morphology. MitoTracker-based imaging revealed that *ITPR1* knockdown significantly reduced mitochondrial fragmentation, as evidenced by a decreased proportion of fragmented mitochondria (Supplementary Figure S2E,F). Finally, Western blot analysis showed a reduction in autophagy- and mitophagy-related markers in ITPR1-deficient cells (Figure 4M), suggesting impaired mitochondrial quality control. Collectively, these results demonstrate that ITPR1 preserves ER–mitochondria Ca^2+^ coupling and maintains mitochondrial dynamics by promoting DRP1 activation and mitochondrial fission, thereby sustaining mitophagy and mitochondrial homeostasis [28].

### 3.4. ITPR1 Preserves Mitochondrial Integrity and Prevents ROS-Mediated Intrinsic Apoptosis in GBM Cells

**Figure 5:**
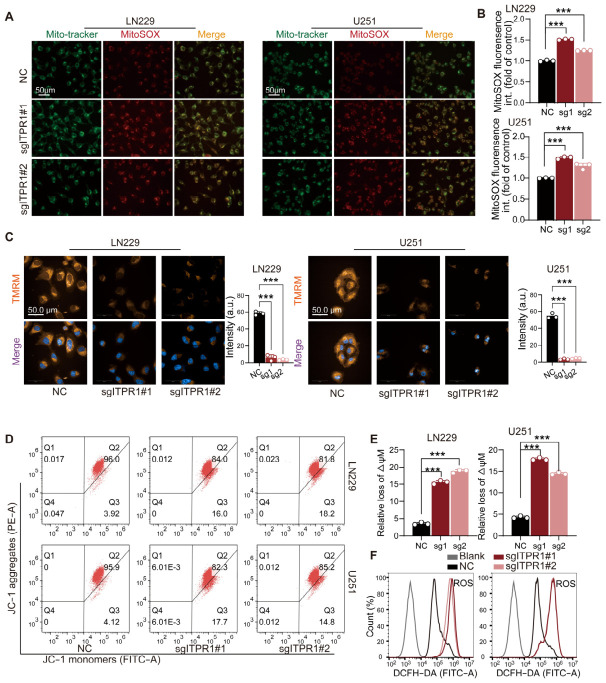
*ITPR1* knockdown induces mitochondrial depolarization, reactive oxygen species (ROS) accumulation, and intrinsic apoptosis in GBM cells. (**A**) Representative images of mitochondrial ROS measurement by MitoTracker and MitoSOX staining (scale bar: 50 μm). (**B**) Quantification of mitochondrial ROS in different groups of GBM cells. (**C**) Quantification of mitochondrial membrane potential in LN229 and U251 cells under control (NC) and *ITPR1* knockdown (sgITPR1#1 and sgITPR1#2) conditions using tetramethylrhodamine methyl ester (TMRM) staining. (**D**) Representative images of mitochondrial membrane potential (ΔΨm) analysis by flow cytometry using JC-1 staining in NC and sgITPR1 cells. (**E**) Quantification of mitochondrial membrane potential (ΔΨm) using JC-1 staining in NC and sgITPR1 cells. (**F**) Intracellular ROS levels measured by DCFH-DA demonstrating elevated ROS accumulation after *ITPR1* knockdown. (*** *p* < 0.001).

Given that ITPR1 mediates Ca^2+^ transfer at ER–mitochondria contact sites, we next examined whether *ITPR1* knockdown affects mitochondrial function in GBM cells. We first assessed mitochondrial reactive oxygen species (ROS) production using MitoSOX staining. As shown in Figure 5A,B, *ITPR1* knockdown markedly increased mitochondrial ROS levels compared with control cells, indicating enhanced oxidative stress at the mitochondrial level.

We then evaluated mitochondrial membrane potential (ΔΨm) using two independent approaches. TMRM staining revealed a significant reduction in ΔΨm in ITPR1-deficient cells (Figure 5C), consistent with mitochondrial depolarization. This finding was further confirmed by JC-1 staining followed by flow cytometry analysis, which showed a shift from red to green fluorescence, indicative of loss of mitochondrial membrane potential (Figure 5D,E). In addition to mitochondrial ROS, total intracellular ROS levels were also elevated upon *ITPR1* knockdown, as measured by DCFH-DA staining (Figure 5F), further supporting the presence of oxidative stress. These findings collectively suggest that loss of ITPR1 impairs mitochondrial integrity, promotes oxidative stress, and triggers mitochondria-dependent apoptosis in GBM cells.

### 3.5. DRP1 Mediates the Pro-Survival and Mitochondrial Regulatory Effects of ITPR1 in GBM Cells

**Figure 6:**
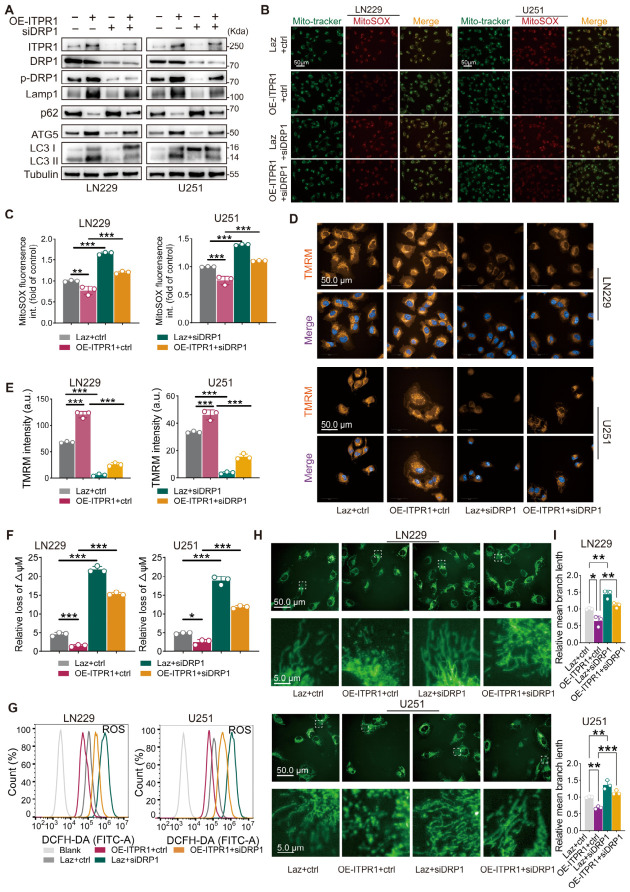
DRP1 is required for ITPR1-mediated regulation of mitochondrial function and autophagy in GBM cells. (**A**) Western blot analysis showing autophagy markers in control, ITPR1-overexpressing, DRP1-knockdown, and DRP1-knockdown ITPR1-overexpressing cells. (**B**) Representative images of mitochondrial reactive oxygen species (ROS) measurement by MitoTracker and MitoSOX staining in the indicated cell groups (scale bar: 50 μm). (**C**) Quantification of mitochondrial ROS measurement by MitoTracker and MitoSOX staining in the indicated cell groups. (**D**) Representative confocal images of LN229 and U251 cells stained with TMRM under the indicated conditions (Laz+ctrl, OE-ITPR1+ctrl, Laz+siDRP1, OE-ITPR1+siDRP1) (Scale bar: 50 μm). (**E**) Quantification of TMRM fluorescence intensity as an indicator of mitochondrial membrane potential. (**F**) Quantification of mitochondrial membrane potential (ΔΨm) levels using JC-1 staining in control, ITPR1-overexpressing, DRP1-knockdown and DRP1-knockdown ITPR1-overexpressing cells. (**G**) Representative histograms of cellular ROS levels detected by DCFH-DA in different cell groups. (**H**) Representative confocal images of mitochondrial morphology in LN229 and U251 cells stained with MitoTracker Green under the indicated conditions (Laz+ctrl, OE-ITPR1+ctrl, Laz+siDRP1, OE-ITPR1+siDRP1). Insets show enlarged views of mitochondrial structures. Scale bars: 50 μm (upper panels) and 5 μm (lower panels). (**I**) Quantification of mitochondrial mean branch length. Data are presented as relative mean branch length normalized to the control. (* *p* < 0.05, ** *p* < 0.01, *** *p* < 0.001).

To determine whether DRP1 functions downstream of ITPR1, we established ITPR1-overexpressing LN229 and U251 cell lines using a CRISPR activation system and silenced DRP1 using specific siRNA. As shown in Figure 6A, DRP1 knockdown alone reduced the levels of autophagy-related proteins compared with control cells. In contrast, *ITPR1* overexpression increased autophagy marker expression, and this effect was partially attenuated by concurrent DRP1 knockdown, suggesting that DRP1 is required for ITPR1-mediated autophagy regulation.

We next assessed mitochondrial oxidative stress. MitoSOX staining showed that *ITPR1* overexpression significantly reduced mitochondrial ROS levels, whereas DRP1 knockdown restored ROS accumulation in ITPR1-overexpressing cells (Figure 6B,C). We further evaluated mitochondrial membrane potential (ΔΨm). TMRM staining revealed that *ITPR1* overexpression enhanced mitochondrial membrane potential, while DRP1 knockdown markedly reduced ΔΨm and counteracted the effect of ITPR1 (Figure 6D,E). CCCP treatment was included as a positive control for ΔΨm dissipation (Supplementary Figure S3A). This result was further confirmed by JC-1 staining, which showed a corresponding shift indicative of mitochondrial depolarization upon DRP1 knockdown (Figure 6F and Figure S3B). Consistently, total intracellular ROS levels, measured by DCFH-DA, were increased upon DRP1 knockdown (Figure 6G), indicating that DRP1 is required for maintaining redox homeostasis downstream of ITPR1.

Given the central role of DRP1 in mitochondrial dynamics, we next analyzed mitochondrial morphology. MitoTracker staining demonstrated that *ITPR1* overexpression promoted mitochondrial fragmentation, whereas DRP1 knockdown resulted in elongated and interconnected mitochondrial networks (Figure 6H). Quantitative analysis of mean branch length confirmed that DRP1 knockdown significantly increased mitochondrial network connectivity and attenuated the fragmentation induced by *ITPR1* overexpression (Figure 6I and Figure S3C). These results suggest that DRP1 may act as a downstream effector of ITPR1, mediating its regulatory effects on mitochondrial dynamics, redox homeostasis, autophagy, and potentially GBM cell survival and proliferation.

**Figure 7:**
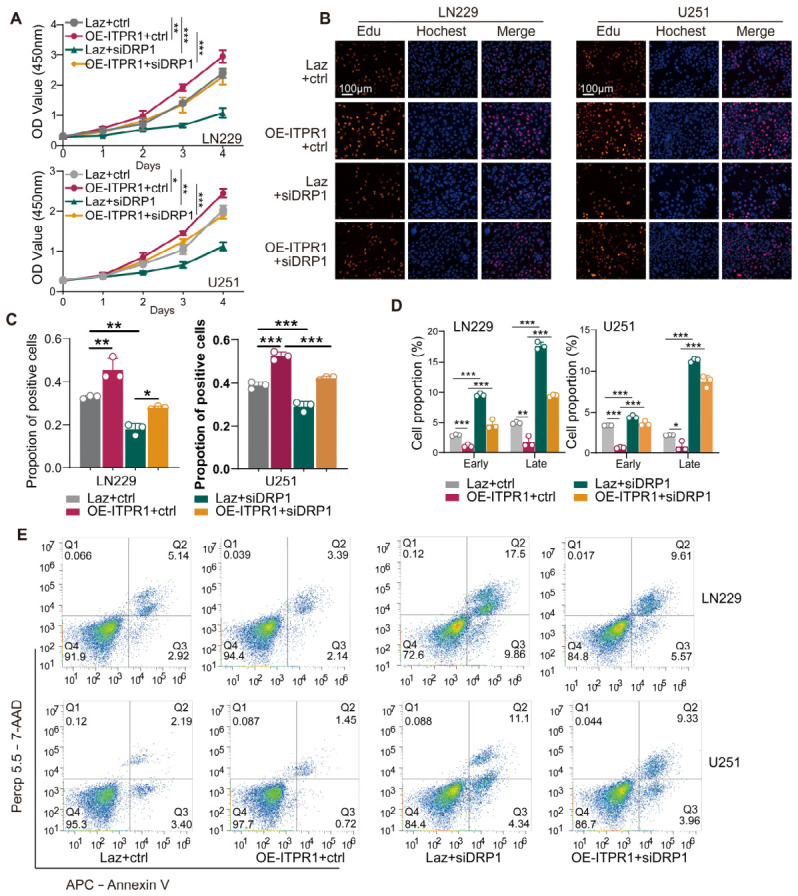
DRP1 knockdown attenuates ITPR1-induced GBM cell proliferation and survival. (**A**) CCK8 assay showing the cell viability in ITPR1-overexpressing and DRP1-knockdown ITPR1-overexpressing cells. (**B**) Representative images of EdU assay in ITPR1-overexpressing and DRP1 knockdown ITPR1-overexpressing cells (scale bar: 100 μm). (**C**) Quantification of cell proliferation measured by the Edu assay in control, ITPR1-overexpressing, and DRP1-knockdown ITPR1-overexpressing cells. (**D**) Quantification of apoptosis in ITPR1-overexpressing and DRP1-knockdown ITPR1-overexpressing cells. (**E**) Representative flow cytometry plots of apoptosis markers (Annexin V/7-AAD staining) in ITPR1-overexpressing and DRP1-knockdown ITPR1-overexpressing cells. (* *p* < 0.05, ** *p* < 0.01, *** *p* < 0.001).

Functionally, DRP1 knockdown partially reversed the enhanced proliferative capacity and reduced apoptotic rate observed in ITPR1-overexpressing cells. CCK8 and EdU assays demonstrated that the pro-proliferative effect of ITPR1 was significantly attenuated upon DRP1 knockdown (Figure 7A–C). Moreover, flow cytometric analysis revealed that DRP1 silencing restored apoptosis levels in ITPR1-overexpressing cells (Figure 7D,E).

### 3.6. Modulation of IP_3_R-Mediated Ca^2+^ Signaling by 2-APB Enhances TMZ Efficacy and Suppresses GBM Progression In Vivo

Temozolomide (TMZ), the standard chemotherapeutic agent for GBM, exerts cytotoxic effects primarily through the induction of DNA damage and oxidative stress [29]. Given the role of ITPR1 in regulating mitochondrial redox homeostasis and apoptosis, we hypothesized that pharmacological modulation of IP_3_R-mediated Ca^2+^ signaling might enhance TMZ sensitivity.

**Figure 8:**
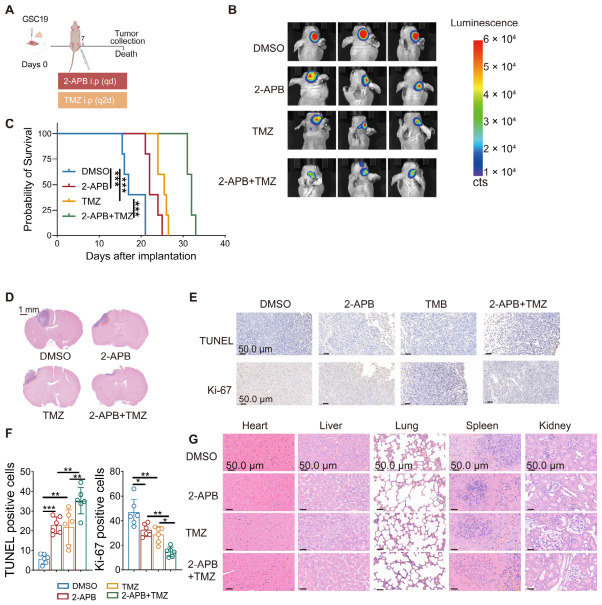
Modulation of IP_3_R-mediated Ca^2+^ signalling by 2-APB enhances TMZ efficacy and suppresses GBM progression in vivo. (**A**) Schematic of orthotopic implantation of GSC19 cells into mice and treatment regimen with 2-APB, TMZ, or their combination (created in BioRender; TAO, M. (2026); https://app.biorender.com/citation/691475822fe169b65998768c, accessed on 14th January 2026). (**B**) Representative bioluminescence imaging of intracranial tumors on Days 7, 14, and 21 showing reduced tumor burden following treatment with 2-APB, TMZ or their combination. (**C**) Kaplan–Meier survival curves showing that combined 2-APB and TMZ treatment confers the greatest survival benefit in GSC19-bearing mice compared to monotherapies. (**D**) Representative H&E staining of mouse brain sections illustrating intracranial tumor suppression by 2-APB, TMZ, and their combination. (**E**) Representative IHC images of TUNEL and Ki-67 staining in tumor tissues following different drug treatments. (**F**) IHC analysis showing TUNEL and Ki-67 staining in tumor tissues treated with different drug treatments. (**G**) H&E staining of organs from nude mice treated with different drug treatments, showing no significant damage to major organs. For in vivo experiments, *n* = 6 mice per group. (* *p* < 0.05, ** *p* < 0.01, *** *p* < 0.001).

To test this hypothesis, we employed 2-aminoethoxydiphenyl borate (2-APB), a well-characterized allosteric modulator of IP_3_Rs that inhibits IP_3_R-mediated Ca^2+^ flux [30]. An orthotopic xenograft model was established by intracranial implantation of GSC19 cells into immunodeficient mice, followed by treatment with 2-APB, TMZ, or their combination (Figure 8A). In vivo bioluminescence imaging revealed that both 2-APB and TMZ monotherapies significantly reduced tumor burden compared with control treatment. Notably, combined administration of 2-APB and TMZ produced a markedly greater suppression of tumor growth (Figure 8B). Consistently, Kaplan–Meier survival analysis demonstrated that combination therapy conferred the most significant survival advantage relative to either agent alone (Figure 8C). Histopathological examination further confirmed reduced intracranial tumor expansion in the combination treatment group (Figure 8D). Immunohistochemical analysis of xenograft tumor sections showed that dual treatment significantly increased apoptotic cell death, as evidenced by enhanced TUNEL staining, while simultaneously decreasing Ki-67 expression, indicating reduced proliferative activity (Figure 8E,F). Importantly, H&E staining of major organs revealed no evident systemic toxicity associated with the combined treatment (Figure 8G).

## 4. Discussion

Emerging evidence has established redox homeostasis as a central determinant of GBM progression and therapeutic response [31]. While ER–mitochondrial communication and DRP1-dependent mitochondrial fission have been implicated in tumor biology, the upstream regulatory mechanisms linking ER Ca^2+^ signaling to mitochondrial quality control remain poorly defined, particularly in a GBM cell state-specific manner [32,33]. Importantly, given the well-established role of ITPR1 in neuronal calcium signaling and cerebellar function, these findings may also have broader implications for understanding redox dysregulation in neurological diseases. Our findings suggest that ITPR1 may position upstream of DRP1-dependent mitochondrial dynamics and that disruption of ER–mitochondria Ca^2+^ coupling might precipitate mitochondrial depolarization, excessive ROS accumulation, and activation of intrinsic apoptosis. Importantly, pharmacological blockade of IP_3_R-mediated Ca^2+^ flux may enhance TMZ efficacy in orthotopic models, suggesting that ER–mitochondria Ca^2+^ signaling may be a redox-centered therapeutic vulnerability in aggressive GBM.

Single-cell transcriptomic analysis revealed preferential enrichment of ITPR1 in MES-like malignant cells, a lineage state strongly associated with inflammatory signaling, invasiveness, recurrence, and resistance to therapy [34]. MES-like cells are known to exhibit heightened oxidative stress tolerance and metabolic plasticity [35]. Our data suggest that ITPR1-mediated Ca^2+^ flux may contribute to this redox-adaptive phenotype by stabilizing mitochondrial function and preventing ROS overaccumulation. The strong association between elevated ITPR1 expression and poor clinical outcome further underscores the clinical relevance of Ca^2+^-regulated redox control in GBM progression. Notably, this study provides evidence supporting a cell state–associated role of ITPR1 in GBM within the mesenchymal subtype, extending beyond general descriptions of ER–mitochondria or DRP1 function.

Mitochondrial dysfunction and oxidative stress are hallmark features of GBM progression, as excessive ROS accumulation contributes to cellular damage and apoptosis [36]. IP_3_R-mediated Ca^2+^ release at ER–mitochondria interfaces directly modulate mitochondrial bioenergetics and ROS production by stimulating dehydrogenase activity and oxidative phosphorylation [37]. Our findings demonstrate that *ITPR1* knockdown disrupts ER–mitochondria proximity, reduces mitochondrial Ca^2+^ uptake, and compromises mitochondrial membrane potential. By promoting mitophagy, ITPR1 ensures the removal of damaged mitochondria, preventing ROS overproduction and limiting intrinsic apoptosis.

Mitochondrial fission and mitophagy are integral to mitochondrial quality control and redox regulation [38]. DRP1-dependent fission facilitates segregation and removal of damaged mitochondria, thereby limiting ROS amplification and oxidative injury [39]. Although DRP1 has been implicated in glioma progression, the direct upstream Ca^2+^-dependent regulators of DRP1 activity in GBM remain largely unknown [33]. While our co-immunoprecipitation and mass spectrometry data support an interaction between ITPR1 and DRP1, it remains to be determined whether this association reflects a direct physical interaction or is mediated through a larger protein complex at the ER–mitochondria interface. Further studies will be required to resolve the precise molecular nature of this interaction. Our study provides evidence suggesting that ITPR1 may function as an upstream regulator of DRP1 activation in GBM, functionally coupling ER–mitochondria Ca^2+^ transfer to DRP1-driven mitochondrial fission. Loss of ITPR1 impairs DRP1 localization, reduces mitophagy marker expression, and results in the accumulation of structurally abnormal mitochondria with diminished membrane potential.

Excessive mitochondrial ROS may act as an inducer of intrinsic apoptosis through cytochrome c release and caspase activation [40]. Our data show that *ITPR1* knockdown induces mitochondrial depolarization, increases mitochondrial ROS, and activates caspase-dependent apoptosis. These results support a model in which ITPR1 acts as a critical signaling hub that integrates ER–mitochondria Ca^2+^ coupling, mitochondrial turnover, and redox buffering capacity to promote GBM cell survival. Unlike previous studies that separately described ER–mitochondria contact or DRP1 activity, our work establishes a mechanistically and functionally coherent ITPR1-DRP1 signaling axis with direct relevance to mitochondrial redox homeostasis and therapeutic response in GBM.

TMZ exerts cytotoxic effects in part through the induction of oxidative stress and mitochondrial dysfunction [41]. However, cancer cells often develop adaptive mechanisms to mitigate ROS accumulation, thereby limiting therapeutic efficacy [42]. Consistent with this concept, our in vivo results show that pharmacological modulation of IP_3_R-related Ca^2+^ signaling by 2-APB may enhance the antitumor efficacy of TMZ, leading to increased tumor apoptosis and reduced proliferative activity. 2-APB is a cell-permeable boron-containing small molecule that has been widely used as a pharmacological tool to investigate intracellular Ca^2+^ signaling pathways. Previous studies indicate that 2-APB can modulate the activity of IP_3_Rs, including ITPR1, through an allosteric mechanism that reduces channel responsiveness to IP_3_ stimulation rather than directly competing for the IP_3_ binding site [43,44]. Through this mechanism, 2-APB has been broadly used to experimentally modulate endoplasmic reticulum Ca^2+^ release mediated by IP_3_ receptors. Importantly, 2-APB is not a highly selective inhibitor and has well-documented off-target effects on several Ca^2+^ signaling pathways, which should be considered when interpreting its therapeutic effects. For example, 2-APB can modulate store-operated Ca^2+^ entry (SOCE) in a concentration-dependent manner, enhancing SOCE at low concentrations while inhibiting it at higher concentrations [45,46]. In addition, 2-APB has been reported to interact with multiple transient receptor potential (TRP) channels, including inhibition of TRPC3/6 and TRPM2/7, as well as activation of TRPV1/2/3 at higher concentrations [47,48]. Other reported effects include modulation of gap junction conductance and mild intracellular acidification under certain experimental conditions [49,50].

Despite these limitations in specificity, 2-APB remains one of the most commonly used pharmacological tools for probing IP_3_R-associated Ca^2+^ signaling [51]. In addition, modulation of IP_3_R-mediated Ca^2+^ signaling by 2-APB has also been explored in neurological disease models, further supporting the conserved role of ITPR1-related signaling in redox regulation across disease contexts [14,51,52]. Taken together, while our findings support an important role for ITPR1-associated Ca^2+^ signaling in mediating TMZ resistance, our genetic data provide specific support for ITPR1 involvement, whereas the pharmacological effects observed with 2-APB should be interpreted in the context of its broader Ca^2+^ channel–modulating activity. Therefore, our results are best viewed as evidence that disruption of IP_3_R-related Ca^2+^ signaling can sensitize glioblastoma to TMZ, rather than definitive evidence of strictly ITPR1-specific pharmacological inhibition.

Although these findings, several limitations should be acknowledged. Both public datasets and in vivo models were used, the sample size of clinical specimens remains relatively limited, which may affect the generalizability of the conclusions. Furthermore, while our data support a functional link between ITPR1 and DRP1, the precise molecular mechanism underlying their interaction requires further investigation, particularly to distinguish direct binding from complex-mediated regulation at ER–mitochondria contact sites. Third, although 2-APB was used as a pharmacological modulator of IP_3_R-mediated Ca^2+^ signaling, its known off-target effects on other calcium channels should be taken into account when interpreting the therapeutic results. Finally, additional studies using more selective inhibitors or genetic models will be necessary to validate the translational potential of targeting ITPR1 in GBM.

In conclusion, our study provides compelling evidence that ITPR1 plays a critical role in regulating mitochondrial ROS and oxidative stress in GBM. By maintaining mitochondrial dynamics and controlling ROS production, ITPR1 supports GBM progression and therapeutic resistance. Targeting ITPR1-mediated Ca^2+^ signaling as well as the recruitment and activation of DRP1 may provide a rationale for developing therapeutic strategies targeting ITPR1-mediated Ca^2+^ signaling in GBM. These findings further suggest that ITPR1-mediated ER–mitochondrial signaling represents a conserved mechanism that may be relevant not only to cancer progression but also to neurological disease.

## 5. Conclusions

Our study identifies that ITPR1 sustains ER–mitochondria Ca^2+^ coupling and preserves mitochondrial integrity by facilitating DRP1 activation, mitochondrial recruitment, and mitophagy, thereby limiting ROS accumulation and intrinsic apoptosis. Importantly, pharmacological inhibition of IP_3_R-mediated Ca^2+^ flux using 2-APB enhances the anti-tumor efficacy of temozolomide. These findings highlight ITPR1 inhibition as a promising therapeutic strategy to enhance treatment response in GBM. 

## Figures and Tables

**Table 1:** Correlation analysis of ITPR1 protein expression in glioma tissues and its association with clinicopathological features.

Characteristics	Total	ITPR1 Expression N (%)		χ^2^/t	*p* Value
		Low	High		
n	175	102	73		
Gender				0.459	0.498
Male	93	52 (29.7%)	41 (23.4%)		
Female	82	50 (28.6%)	32 (18.3%)		
Age	175	46.775 ± 15.71	47.438 ± 14.759	−0.282	0.778
Grade				4.326	0.038 *
Low	100	65 (37.1%)	35 (20%)		
High	75	37 (21.1%)	38 (21.7%)		
Recurrence				4.478	0.034 *
No	123	78 (44.6%)	45 (25.7%)		
Recurred	52	24 (13.7%)	28 (16%)		
OS				3.487	0.061
Live	110	70 (40%)	40 (22.9%)		
Dead	65	32 (18.3%)	33 (18.9%)		
Median survival (months)		103	82	13.46	<0.001 ***

* *p* < 0.05, *** *p* < 0.001.

## Data Availability

The bulk RNA-seq data in this study were obtained from the TCGA and CGGA databases. Moreover, the annotated scRNA-seq data were downloaded via cellxgene at (https://cellxgene.cziscience.com/collections/999f2a15-3d7e-440b-96ae2c806799c08c (accessed on 10 April 2026)). The corresponding author can provide all other data to support this study upon reasonable request.

## Supplementary Materials

The following supporting information can be downloaded at: https://www.mdpi.com/article/10.3390/antiox15050550/s1, Supplementary Table S1: Meta-module gene lists. Supplementary Table S2: Sequences of sgRNA and siRNA used in the study. Supplementary Table S3: Liquid chromatography–tandem mass spectrometry analysis of the ITPR1 and IgG antibody in LN229 cells. Supplementary Figure S1: Single-cell analysis of neoplastic and non-neoplastic clusters and ITPR1 expression in GBM samples; Supplementary Figure S2: Mitochondrial morphology and ER–mitochondria colocalization in LN229 cells under ITPR1 knockdown; Supplementary Figure S3: Assessment of mitochondrial membrane potential and morphology in LN229 and U251 cells with ITPR1 and DRP1 perturbations.

## Abbreviations

2-APB	2-aminoethoxydiphenyl borate
AC-like	astrocyte-like
CGGA	Chinese Glioma Genome Atlas
cryo-ET	cryo-electron tomography
cryo-FIB	cryo-focused ion beam
DEGs	differentially expressed genes
ER	endoplasmic reticulum
FC	fold change
GBM	glioblastoma
GSCs	glioma stem cells
IHC	immunohistochemistry
IP	immunoprecipitation
IP_3_R	inositol 1,4,5-trisphosphate receptor
LC-MS/MS	liquid chromatography–tandem mass spectrometry
LDH	lactate dehydrogenase
MAMs	mitochondria-associated membranes
MES	mesenchymal
MES-like	mesenchymal-like
MMP	mitochondrial membrane potential
NPC-like	neural progenitor cell-like
OD	optical density
OPC-like	oligodendrocyte progenitor cell-like
ROS	reactive oxygen species
scRNA-seq	single-cell RNA sequencing
TCGA	The Cancer Genome Atlas
TEM	transmission electron microscopy
TMZ TRP	temozolomide transient receptor potential
VDACs	voltage-dependent anion channels
