# Supplementary material for: ITPR1 Maintains Mitochondrial Redox Homeostasis to Drive Glioblastoma Progression Through Recruitment and Activation of DRP1
Source: Antioxidants (Basel). 2026 Apr 26;15(5):550. doi: 10.3390/antiox15050550 (PMC13203755; doi:10.3390/antiox15050550)
Supplement: Supplementary file 1 [file antioxidants-15-00550-s001.zip › Supplementary Figure S1-S3.pdf]

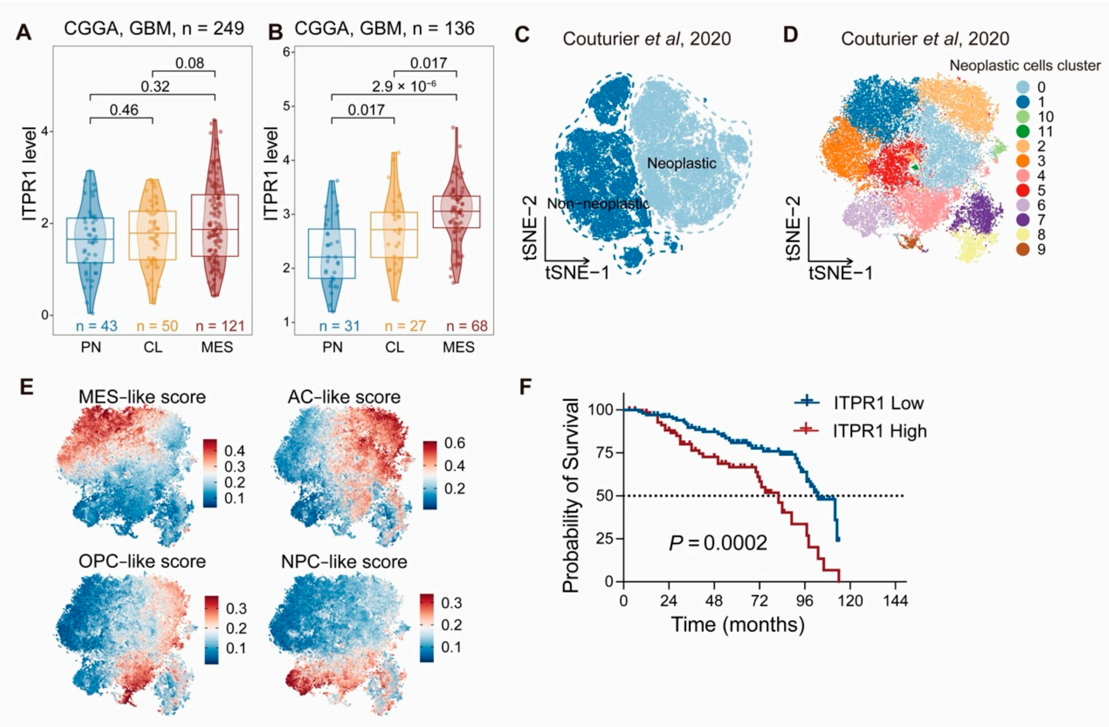

Supplementary Figure S1. Single-cell analysis of neoplastic and non-neoplastic clusters and ITPR1 expression in GBM samples. (**A**) t-SNE plot depicting non-neoplastic and neoplastic clusters in GBM samples. (**B**) t-SNE plot showing the subclusters of neoplastic GBM cells. (**C**) t-SNE plots showing the expression of MES-like, AC-like, OPC-like, and NPC-like malignant cell state marker genes calculated by AUCell in neoplastic GBM cells. (**D–E**) Violin plot showing ITPR1 expression of GBM patients across different neoplastic subcellular clusters defined by the malignant meta-program subtypes in the CGGA693 cohort (**D**) and CGGA325 cohort (**E**). (**F**) Kaplan–Meier analysis of overall survival in the entire glioma cohorts stratified by ITPR1 expression. Patients with high ITPR1 expression showed significantly shorter overall survival than those with low ITPR1 expression. Statistical significance was determined using the log-rank test.

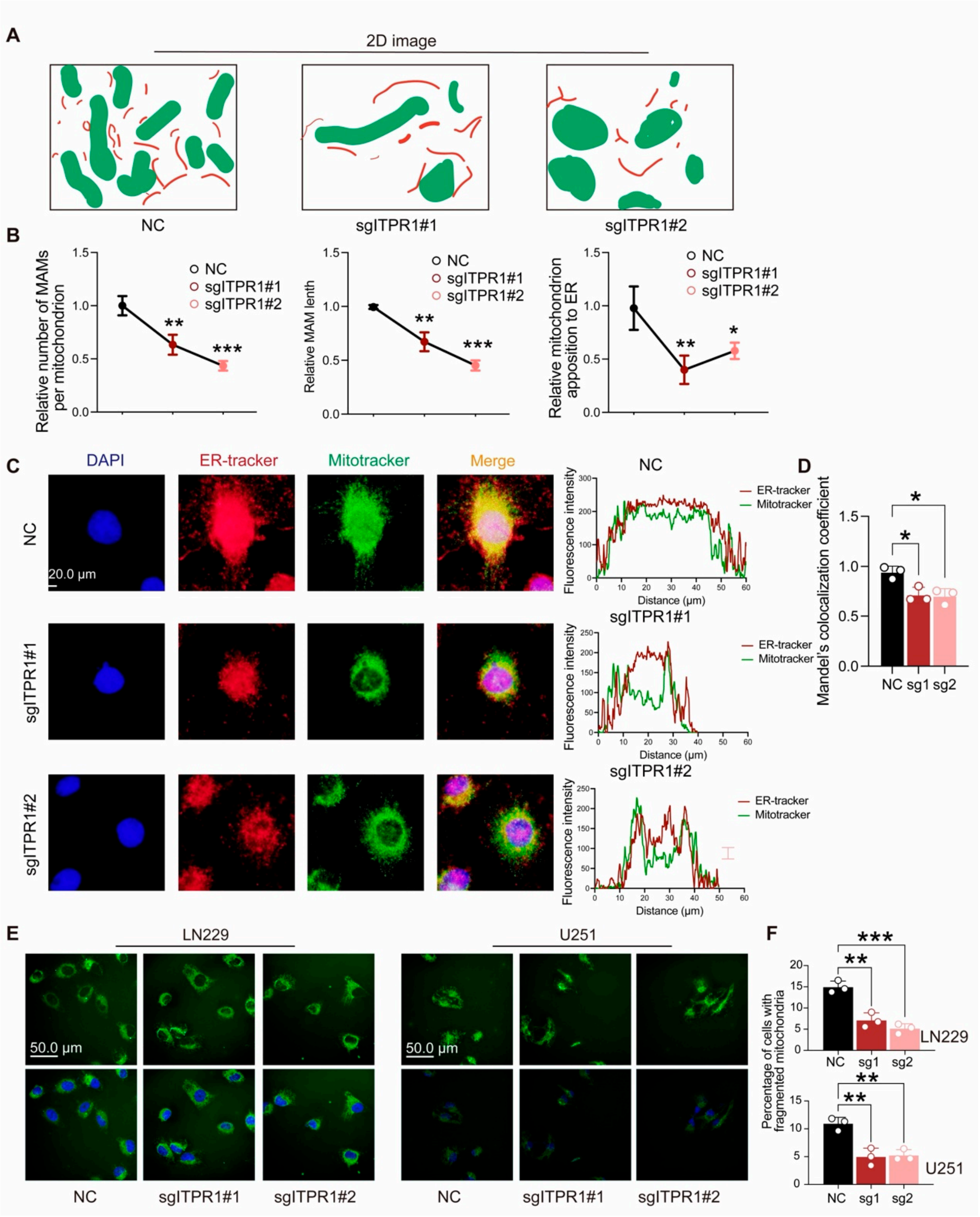

Supplementary Figure S2. Mitochondrial morphology and ER–mitochondria colocalization in LN229 cells under ITPR1 knockdown. (**A**) Representative 2D images in different groups of LN229 cells. (**B**) Quantitative statistical analysis of MAM structures in different groups of LN229 cells. (**C**) Representative confocal images of live GBM cells stained with ER-Tracker (red) and MitoTracker (green) under control and ITPR1 knockdown conditions. (**D**) Quantification of ER–mitochondria colocalization using Manders' overlap coefficient (M1 and M2). (**E**) Representative confocal images of mitochondrial morphology in LN229 and U251 cells stained with MitoTracker Green under control (NC) or ITPR1 knockdown (sgITPR1#1 and sgITPR1#2) conditions (scale bar, 50 μm). (**F**) Quantification of the percentage of cells with fragmented mitochondria. (*$p < 0.05$, **$p < 0.01$, ***$p < 0.001$)

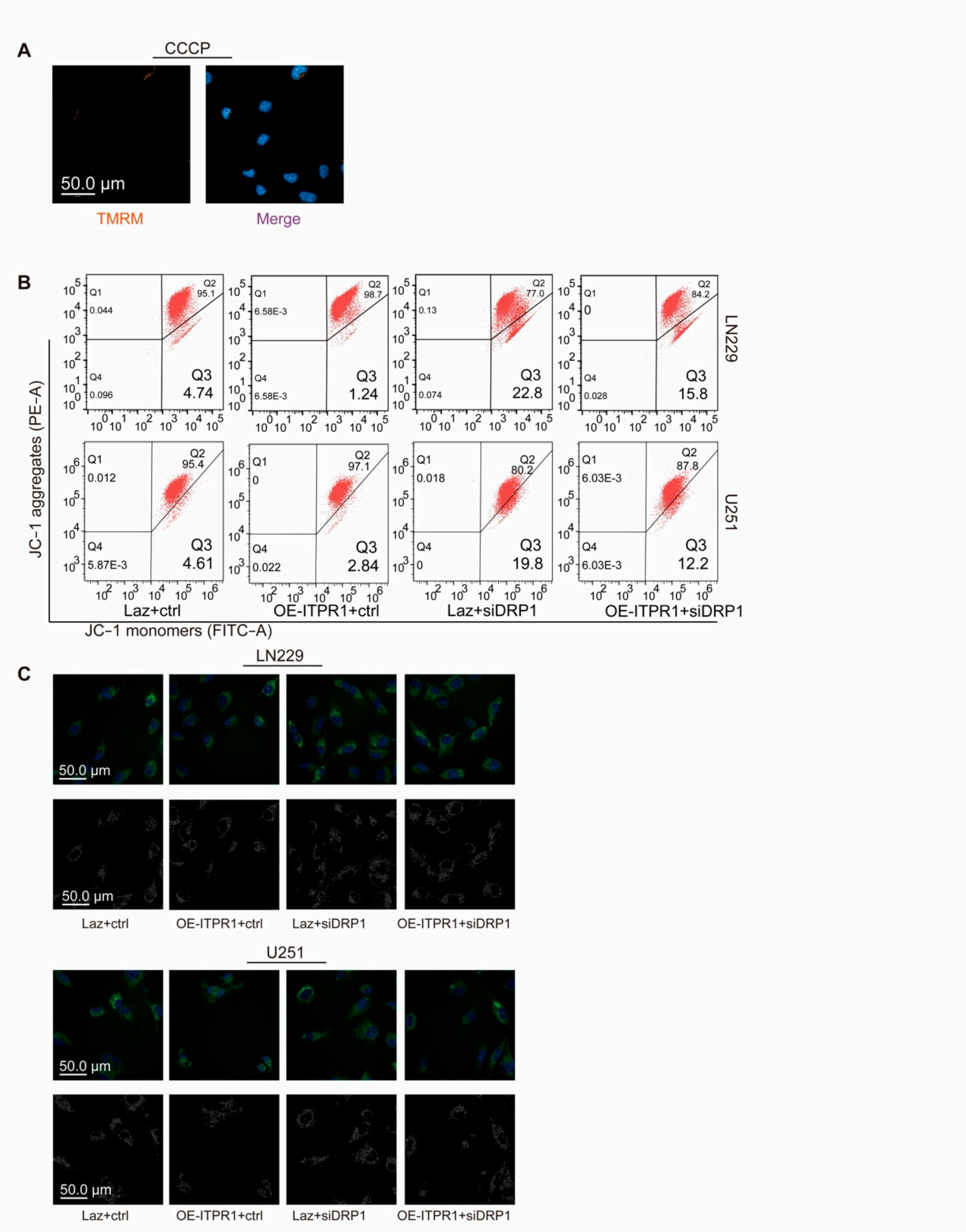

Supplementary Figure S3. Assessment of mitochondrial membrane potential and morphology in LN229 and U251 cells with ITPR1 and DRP1 perturbations. (**A**) Tetramethylrhodamine methyl ester (TMRM) staining for mitochondrial membrane potential (ΔΨm) in cells treated with CCCP, serving as a positive control for complete ΔΨm loss. (**B**) Representative flow cytometry plots of mitochondrial membrane potential (ΔΨm) using JC-1 staining in ITPR1-overexpressing and DRP1-knockdown ITPR1-overexpressing cells. (**C**) Representative confocal images of LN229 and U251 cells under the indicated conditions (Laz+ctrl, OE-ITPR1+ctrl, Laz+siDRP1, OE-ITPR1+siDRP1). Cells were stained as indicated to visualize mitochondrial morphology and cellular structure (scale bar: 50 μm). Lower panels show the corresponding mitochondrial signal to highlight network organization.
