# Supplementary material for: ITPR1 Maintains Mitochondrial Redox Homeostasis to Drive Glioblastoma Progression Through Recruitment and Activation of DRP1
Source: Antioxidants (Basel). 2026 Apr 26;15(5):550. doi: 10.3390/antiox15050550 (PMC13203755; doi:10.3390/antiox15050550)
Supplement: Supplementary file 1 [file antioxidants-15-00550-s001.zip › Supplementary Table S2.pdf]

Supplementary Table S2. Sequences of sgRNA and siRNA used in the study.

**shRNA / siRNA**	**5'-3'**
sgITPR1#1 (CRISPR i)	CAAGCCCCATTCACAGACTG
sgITPR1#2 (CRISPR i)	GAGGCGGGCATATTTCACGG
OE-ITPR1 (CRISPR a)	ATATAGGCCGGGAAGCTCCC
ctrl-siRNA sense	UUCUCCGAACGUGUCACGUTT
ctrl-siRNA anti-sense	ACGUGACACGUUCGGAGAATT
siDRP1 sense	GCUACUUUACUCCAGCUUATT
siDRP1 anti-sense	UAAGCUGGAGUAAAGUAGCTT
